# Impact of Hurricane Sandy on Hospital Admissions 2 Years Later

**DOI:** 10.1093/geroni/igab046.906

**Published:** 2021-12-17

**Authors:** Laura Sands, Pang Du, Quyen Do, Yunnan Xu, Rachel Pruchno

**Affiliations:** 1 Virginia Tech, Blacksburg, Virginia, United States; 2 Virginia Tech, Virginia Tech, Virginia, United States; 3 Rowan University, Stratford, New Jersey, United States

## Abstract

Disaster exposure is often followed by acute illness and injuries requiring hospital admission in the weeks after the disaster. It is not known whether disaster exposure is associated with hospitalization in the years after the disaster. We examined the extent to which disaster exposure is associated with hospitalization two years after Hurricane Sandy. The analyses fill a gap in our understanding of long-term physical health consequences of disaster exposure by identifying older adults at greatest risk for hospitalization two years after disaster exposure. Older adults (n=909) who participated in a longitudinal panel study provided data before and after Hurricane Sandy. These data were linked with Medicare inpatient files to assess the impact of Hurricane Sandy on hospital admissions after the post-hurricane interview. Those who reported experiencing a lot of fear and distress in the midst of Hurricane Sandy were at an increased risk of being hospitalized in the second or third years after the hurricane [Hazard Ratio=1.81 (1.15 – 2.85)]. Findings held after controlling for pre-hurricane demographics, social risks, chronic conditions, and decline in physical functioning after the hurricane. These findings are the first to show that disaster exposure increases risk for hospital admissions two years after a disaster, and that older adults’ appraisal of their emotional distress during the disaster has prognostic significance that is not explained by known risks for hospital admissions. The findings suggest that interventions during the storm and after the storm, may reduce long-term health consequences of disaster exposure among older adults.

